# Phytoremediation of electroplating wastewater by vetiver grass (*Chrysopogon zizanoides* L.)

**DOI:** 10.1038/s41598-021-93923-0

**Published:** 2021-07-14

**Authors:** Andhika Puspito Nugroho, Erni Saurmalinda Butar Butar, Ekaputra Agung Priantoro, Lies Sriwuryandari, Zulfa Berliana Pratiwi, Tarzan Sembiring

**Affiliations:** 1 Faculty of Biology, Universitas Gadjah Mada, Yogyakarta, Indonesia; 2 Waste Treatment and Environmental Management Working Group, Research Unit for Clean Technology – Indonesian Institute of Sciences, Bandung, Indonesia

**Keywords:** Pollution remediation, Environmental social sciences

## Abstract

The electroplating industry generates wastewater containing a variety of heavy metals which potentially contaminate water ecosystems. The available and well-known electroplating wastewater treatments are considered as an expensive and less effective method, therefore phytoremediation was used as an alternative friendly solution. This study aims to evaluate the uptake and elimination rate of heavy metals by vetiver (*Chrysopogon zizanoides* L.) on metal-polluted water. Vetiver was planted in artificial electroplating wastewater containing different levels (low, medium, high) of chromium (Cr) and nickel (Ni). Water, roots, and shoots were collected periodically to determine Cr and Ni contents using Atomic Absorption Spectrometry (AAS). Metal accumulation and elimination rate, Bioconcentration Factor (BCF), Biological Absorption Coefficient (BAC), and Translocation Factor (TF) were calculated to evaluate plant’s effectiveness in metal remediation processes. The results showed that vetiver (*C. zizanoides L.*) was able to remove 61.10% Cr and 95.65% Ni on metal-contaminated water. The highest uptake rates for Cr and Ni are 127.21 mg/kg/day and 15.60 mg/kg/day respectively, while the elimination rates for Cr and Ni tend to slow 1.09 mg/kg/day and 12.24 mg/kg/day respectively. Vetiver BCF, BAC, and TF values on Cr and Ni contaminated water were greater than 1, which indicates that vetiver work through phytoextraction and phytostabilization to treat metals. The findings showed that vetiver has promise as a phytoremediation agent thus providing implication for electroplating wastewater treatment.

## Introduction

Wastewater from electroplating industrial activities became major concern due to high variety and concentration of heavy metals^1,2^. This attracted great attention in terms of environmental impact and removal technique^3^. Once heavy metal contaminate the aquatic ecosystem, the metal’s toxicity, carcinogenicity, mutagenicity may adverse the aquatic life^4^. Metal is persisted to the environment^4,5^, and potentially to be biomagnified through the food chain^6–9^.

The discharge of metal ions such as chromium (Cr) and nickel (Ni) into surface water may impact on living organism^10^. Chromium has several oxidation states (− 2 to + 6), Cr (VI) and Cr (III) is the most common form in the environment, Cr (III) is less toxic than Cr (VI)^11–14^. While Ni is one of the plant micronutrients, it helps the formation of enzymes such as urease^15^. However, high Cr and Ni levels in the environment may induce plant toxicity such as chlorosis, necrosis, damage on root cells, wilting, nutritional deficiency, disruption of enzymatic activity and induce reactive oxygen species (ROS)^16–18^. Recent electroplating wastewater treatment is considered as an expensive and less effective method, it could produce secondary pollutants^19^. Therefore it is imperative to evaluate phytoremediation as an easy, inexpensive, and environmentally friendly solution in order to remove heavy metals^20,21^.

Phytoremediation is a green technology to remediate environmental pollutants by employing plants^22–25^. Plantsable to accumulate pollutants including heavy metals through phytoextraction, phytostabilization, rhizodegradation, phytotransformation, phytodegradation, and phytovolatilization processes^26–29^. The ability of plants to accumulate and eliminate high content of heavy metals were needed in phytoremediation processes^16,30^. In order to respond to heavy metal stress in the environment, plants produce chelators and organic acids to bind with toxic metal ions^31–34^. The complex between metal and chelator were sequestrated by cell, so the metal ions were inactivated through compartmentalization in cellular parts of plant^15,35^.

Vetiver (*C. zizanoides L.*) has exhibited the potential for polluted water treatment and phytoremediation^7^. As a perennial grass, it was reported that vetiver was high tolerance and effective to reduce heavy metals in wastewater^7,22,35,36^. Vetiver has a wide range of environmental factor tolerances. Although vetiver is terrestrial plant, it grows rapidly and is able to adapt and grow in water, acid environment and temperature stress^22,26,37,38^. Vetiver has erected and stiff shoots, it has massive, deep, and fast-growing root system^39^. These root systems provide an enormous surface area for vetiver to absorb large amounts of pollutant rather than the other species^6,39^. The morphological character of vetiver indicated that plants were suitable as potential phytoremediation agents. Therefore, this study was aimed to understand the metals uptake and elimination rates by vetiver in order to evaluate vetiver as a potential phytoremediation agent.

## Results and discussion

Phytoremediation of Cr and Ni is shown that heavy metal content in plant growth media were reduced during 28 days remediation by vetiver grass (*C. zizanoides* L*.*). The results showed heavy metal reduction were 61.10% and 95.65% in Cr-A treatment and Ni-A treatment respectively (Table 1). The result was relatively greater than the previous report, where Cr and Ni reduction was about 21% and 38% respectively on Cr and Ni elimination from acid mines^40^. However, during 28 days of metal exposure, vetiver plants were affected by Cr toxicity. It was observed in the first 20 day that the grass loss on water content and turgor pressure, wilting, chlorosis, and root cells disruption. Meanwhile, in Ni treatment, the toxicity symptoms that arose in plants are chlorosis, wilting, and necrosis.

**Table 1:** Cr and Ni levels on vetiver (*C. zizanoides* L.) growth media.

Treatments	Uptake (mg/L)		Elimination (mg/L)	
	D0	D28	D0	D28
**Control**				
Cr	0.0000^a^	0.0000^a^	0.0000^a^	0.0000^a^
Ni	0.0000^a^	0.0000^a^	0.0000^a^	0.0000^a^
**Cr**				
A	50.7741^b^	19.7494^b^	0.0000^a^	0.2292^b^
B	155.2206^c^	86.9938^c^	0.0000^a^	0.5737^c^
C	346.9057^d^	190.3750^d^	0.0000^a^	0.9380^d^
**Ni**				
A	24.7387^b^	1.0767^a^	0.0000^a^	0.2113^b^
B	76.3505^c^	12.6903^b^	0.0000^a^	0.5473^c^
C	156.8409^d^	34.0900^c^	0.0000^a^	0.6923^c^

Information: A (Low concentration); B (Medium concentration); C (High concentration).

Identical letter indicated statistically no significant different (p > 0.05).

Despite the series of toxicity symptoms, the vetiver in this experiment was found to be well adapted, indicated with the growth of roots and new individuals in the Cr and Ni treatment. The ability of plants to absorb large amounts of metals and reduce their toxicity is a key factor for the remediation process. This proves that vetiver has a high potential as phytoremediation agent.

During elimination condition 0.60–0.80% Cr and 0.60–0.89% Ni were released back to the plant growth medium (Table 1). The mechanism is used by plants to reduce heavy metals toxicity by controlling the level of heavy metals accumulation in plant cells. The efflux system consisting of P1B-ATPases and CDF transporter families on roots plasma membrane is involved on this mechanism^41–44^.

**Figure 1:**
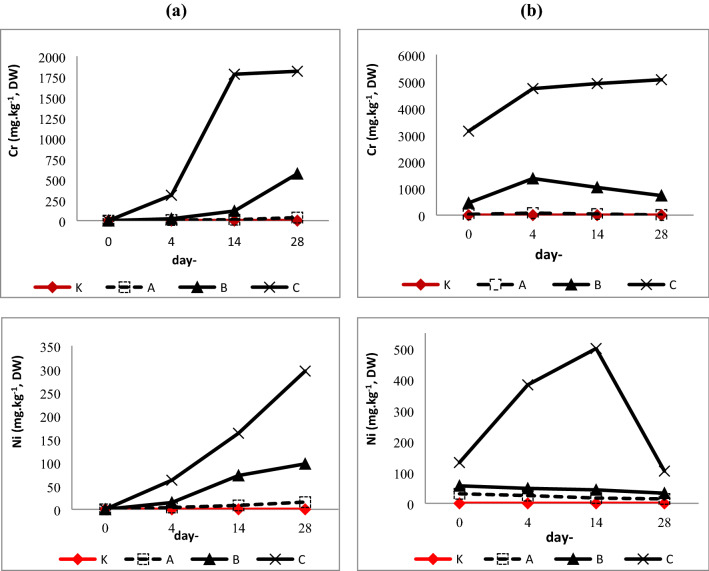
Cr and Ni content in shoots of vetiver (*C. zizanoides* L.): (**a**) uptake condition, (**b**) elimination condition.

The concentration of chromium and nickel content in shoots of vetiver (*C. zizanoides* L.) were increased during 28 days remediation (uptake condition) (Fig. 1a). The metals uptake and translocation in vetiver (*C. zizanoides* L.) occurs gradually over the length of time metal exposure. The highest Cr and Ni accumulation in shoots were 1817.0894 mg/kg, DW and 295.9948 mg/kg, DW respectively. The metals uptake can be affected by metal levels on plant growth medium. High number of biomass was needed to accumulate high metal concentration, therefore NPK and fertilizer were added to support vetiver growth^45^. Chromium and nickel were translocated from vetiver roots to shoots through xilem^46,47^. The translocation process both metals tend to be slow^48,49^ because of Cr tend to be retained on roots vacuole and cell wall^11,50–52^, while more than 50% absorbed nickel were retained in roots cylinder vascular^49,53^.

Metals content in vetiver shoots during elimination condition tend to decrease after reaching the peak of metal accumulation (Fig. 1b). Nevertheless, it was found that metal accumulation in Cr-C treatment still increased until the end of the elimination condition. This indicates that the Cr concentration in Cr-C treatment is too high or not proportional to the slow development of plant biomass. Hence, a longer observation time is needed to understand the elimination of high Cr concentrations in vetiver. Vetiver transfer into non-metal contaminated medium during elimination conditions may support the plant growth. As the plant grows, new biomass will be formed and excess metal will be translocated into younger tissues, therefore the shoots metal content was reduced through the time. Study revealed that chromium and nickel were translocated into older tissue in order to protect young tissues^49,54^.

Rate of metals uptake in vetiver (*C. zizanoides* L.) strongly related with metal concentration in plant growth media. Heavy metals stress induced chelator production which is used by plants to form metals-chelator complex in order to reduce metal toxicity^15^. Metal accumulation in plants was accelerated with the formation of this complex, therefore the rate of metals uptake was increased as the increase of metal content in growth media (Table 2). It was reported that vetiver accumulates chromium better than nickel^55^, as seen on the result, the rate of chromium uptake is higher than nickel.

**Table 2:** Rate of heavy metals uptake and elimination in vetiver (*C. zizanoides* L.).

Samples	Rate of uptake (mg/kg/day)			Rate of elimination (mg/kg/day)		
	4	14	28	4	14	28
Cr-A	1.81	0.79	1.32	− 10.17	− 0.41	1.09
Cr-B	5.61	8.33	20.38	− 199.86	− 33.23	− 5.62
Cr-C	77.06	127.21	64.90	− 728.05	− 221.45	− 115.87
Ni-A	0.97	0.60	0.55	− 2.43	− 0.12	0.04
Ni–B	3.64	5.17	3.48	12.24	3.84	2.30
Ni-C	15.60	11.63	10.57	− 21.76	− 14.61	6.85

Information: A (Low concentration); B (Medium concentration); C (High concentration).

The rate of metals elimination after plants were moved into non-metal contaminated water is extremely slow. Toxic metals can be eliminated by plant through sequestration metal-chelator complex into inactive form, this mechanism is aided by tonoplast antiporter such as cation diffusion facilitator (CDF), cation exchanger (CAX), and magnesium exchangers^15^. Although vetiver has a slow metal elimination rate, it is known to be adaptive in heavy metal stress. Plants with high ability to sequestrate metals were known as good potential phytoremediation^16,22^.

Vetiver (*C. zizanoides* L.) potential as a phytoremediation agent can be determined by some index including bioconcentration factor (BCF), biological absorption coefficient (BAC), and translocation factor (TF). Plants with BAC ≥ 1, BCF ≥ 1, TF ≥ 1 classified as metal hyperaccumulator with strong phytoextraction capacity, while plants with BAC ≥ 1, BCF ≥ 1, TF ≤ 1 classified as metal hyperaccumulator through phytostabilization mechanism^56–58^. Plants were classified as metal hyperaccumulators when they were able to accumulate > 1000 mg/kg, DW metals in their tissues^57,59,60^.

Vetiver (*C. zizanoides* L) able to accumulate Cr > 1000 mg/kg, DW and possess great BAC, BCF, TF values (Table 3). It is suggested that vetiver possess strong phytoextraction and phytostabilization capacity for chromium. On the other hand vetiver was not able to accumulate nickel > 1000 mg/kg, DW, it means vetiver could not be classified as a nickel hyper-accumulator. However, it has high BAC and BCF values (Table 4), so it could be classified as a good potential phytoremediation for nickel with phytostabilization capacity. Together, these results indicate that vetiver (*C. zizanoides* L.) tissues possess a high capacity for heavy metals accumulation, it may grow normally in a metal-polluted environment, and is thus becoming a promising metal pollution tolerant plant species.

**Table 3:** Cr content in root, shoot, and plant growth media after 28 days metal exposure and 28 days moved into freshwater.

Samples	Root (mg/kg, DW)	Shoot (mg/kg, DW)	Water (mg/kg)	BCF	BAC	TF
**Uptake condition**						
Cr-K	33.91^a^ ± 1.47	0.00^a^ ± 0.00	0.13			
Cr-A	1776.62^b^ ± 9.87	36.97^b^ ± 0.19	19.75	89.96	1.87	0.02
Cr-B	2205.82^c^ ± 7.33	570.59^c^ ± 1.33	86.99	25.36	6.56	0.26
Cr-C	3173.70^d^ ± 54.05	1817.09^d^ ± 4.06	190.38	16.67	9.54	0.57
**Elimination condition**						
Cr-K	43.61^a^ ± 1.08	0.00^a^ ± 0.00	0.00			
Cr-A	668.84^b^ ± 7.65	6.51^b^ ± 0.94	0.23	2918.16	28.41	0.01
Cr-B	1734.06^c^ ± 0.63	730.19^c^ ± 3.95	0.57	3022.60	1268.80	0.42
Cr-C	2468.07^d^ ± 24.47	5061.57^d^ ± 13.82	0.94	2631.21	5396.13	2.05

Information: K (Control); A (Low concentration); B (Medium concentration); C (High concentration).

Identical letter indicated statistically no significant different (p > 0.05).

**Table 4:** Ni content in root, shoot, and plant growth media after 28 days metal exposure and 28 days moved into freshwater.

Samples	Root (mg/kg, DW)	Shoot (mg/kg, DW)	Water (mg/kg)	BCF	BAC	TF
**Uptake condition**						
Ni-K	22.30^a^ ± 0.25	0.00^a^ ± 0.00	0.00			
Ni-A	1279.20^b^ ± 5.20	15.49^b^ ± 1.45	1.08	1188.08	14.39	0.01
Ni–B	1563.01^c^ ± 10.18	97.35^c^ ± 0.41	12.69	123.166	7.67	0.06
Ni-C	16,533.21^d^ ± 9.48	295.99^d^ ± 0.32	34.09	48.4954	8.68	0.18
**Elimination condition**						
Ni-K	8.60^a^ ± 0.37	0.00^a^ ± 0.00	0.00			
Ni-A	625.03^b^ ± 3.57	14.28^b^ ± 0.66	0.21	2958.73	68.05	0.02
Ni–B	576.00^c^ ± 2.65	32.82^c^ ± 0.66	0.55	1052.53	59.97	0.06
Ni-C	998.60^d^ ± 8.75	104.29^d^ ± 0.35	0.69	1442.43	150.64	0.10

Information: K (Control); A (Low concentration); B (Medium concentration); C (High concentration).

Identical letter indicated statistically no significant different (p > 0.05).

Heavy metals became an inert form inside the plant cells^15^, to become the part of plant biomass. Disposal strategies for metal rich senescent leaves are also needed in order to prevent secondary metals contamination issues^53^. Pyrolysis, gasification, incineration, and volume reduction processes such as composting and compacting are some of the methods that are usually used to manage metal rich plant biomass^53^.

## Conclusions

Based on the results we concluded that *C. zizanoides L.* potentially used as a phytoremediation agent for electroplating wastewater treatment. It significantly reduced Cr and Ni levels on electroplating wastewater. Metal accumulation and elimination rate by *C. zizanoides L* strongly indicated the capability *C. zizanoides L.* to uptake and detoxify metals on its biomass. Furthermore, great BAC, BCF, and TF values suggest that *C. zizanoides L.* are capable of processing process phytoextraction and phytostabilization during remediation processes.

## Method and materials

### Preliminary

Tools experiments were soaked with HNO_3_ 0.1 M overnight and rinsed with distilled water. Vetiver which adapted to water-growth media was selected. To obtain a solitary plant, vetiver were separated from the clump and moved to another container to be acclimatized. Electroplating wastewater was made artificially using K_2_Cr_2_O_7_ and NiSO_4_·6H_2_O. Chromium concentrate solution was made with diluting 5.66 g K_2_Cr_2_O_7_ in a 1000 mL reaction flask, while nickel standard solution was made by diluting 4.48 g NiSO_4_**.**6H_2_O in a 1000 mL aquabidest. Different levels concentration of chromium (Cr) and nickel (Ni) as artificial wastewater were made by dilution of concentrate solution.

### Plant materials

Vetiver used in the experiment is nursery and cultivated in Green house, a collection of Laboratory for Waste and Wastewater management- Research Unit for Clean Technology—Indonesian Institute of Sciences. After the experiment all vetiver materials including samples would be ignite and the ash collected in a special container before deposit in hazardous waste deposit bureau belong to national government. The use of the plant fulfills the law according to the letter of permission no: B-390/IV/DI.01.03/5/2021 from The Secretariat of Scientific Authority for Biodiversity—Indonesian Institute of Sciences.

### Heavy metal exposure

Artificial wastewater was used as plant growth media. *C. zizanoides L.* were moved to metal-contaminated growth media indicated that the day-0 of metal exposure. Plants were exposed to metal for 28 days indicating uptake condition, then plants were moved into non-metal contaminated growth media for 28 days indicating the elimination condition. To minimize the loss of water due to evapotranspiration, distilled water was added into plant growth media until reaches the initial volume of water growth media. Fertilizer (6.20 g NPK and 2.06 g urea diluted in 100 mL water) and compost leachates (5 g) were added on the day-0 both in uptake and elimination conditions to meet plants nutrition.

### Sampling

**Figure 2:**
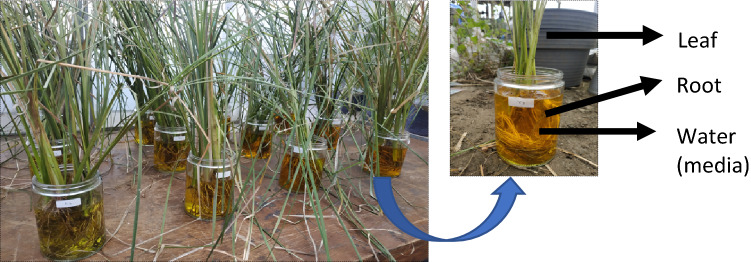
Sampling vetiver site.

We collected samples consisting of water (media), leaf, and root samples. Sampling site was shown on Fig. 2. Water samples were collected at the day-0 and day-28 both in uptake and elimination conditions for metal content and *Chemical Oxygen Demand* (COD) determination. Leaf samples were collected atday-0, 4, 14, 28 of both in uptake and elimination conditions, for metal content determination. Root samples were also taken at day-0 and day-28 both in uptake and elimination conditions for the determination of metal content. Water acidity (pH), water temperature, humidity, and air temperature were collected as additional supporting data.

### Samples measurement

The water samples were filtered using Whatman 42 (pore size 2.5 µm) then 50 mL of water were digested using 5 mL HNO_3_ in 250 mL Erlenmeyer which was covered by glass funnel. Samples were heated slowly until reaching a clear color and the remaining volume is about 15–20 ml. Water-digested samples were diluted and homogenized with 50 mL distilled water before measurement. The plant samples were dried in an oven at 70 °C to a constant weight. The dried plant tissues were subsequently weighed and ground into powder. Two (2) mL of HNO_3_ 65% were added to 0.2 g of plant samples in a 100 ml Erlenmeyer and stirred. Then 1.6 mL of H_2_O_2_ 33% were carefully added and slightly stirred after the addition, followed by heating the sample on a hot plate, then a strong effervescence would be produced. At about 7–8 min the brown fumes produced were less dense and it allowed for cooling. A slightly yellow solution was obtained, filtered and washed with 5 mL of (1:1) HCl (density 1.18 g/mL) and diluted with 25 mL of distilled water. The heavy metals contents of all samples were measured using Atomic Absorption Spectrometry (Agilent Technology). *Chemical Oxygen Demand* (COD) was determined using Spectrophotometer UV–Vis (Agilent Technology). 1.5 mL of water sample for COD measurement were added into reagent which contain the mixture of 750 mL H_2_SO_4_, 25 mL orthophosphate, 15 g Cr_2_O_7_, 10gr Ag_2_SO_4_, then reflux for 2 h at the temperature 150 °C.

### Analysis

The results were tabulated in Microsoft Excel 2016. Statistical analysis independent t-test (p < 0.05) were conducted using software IBM SPSS *Statistics* v.16. Bioconcentration Factor (BCF) as metal concentration ratio of plant roots to the water, Biological Absorption Coefficient (BAC) as ratio heavy metal content in plant and the water, and Translocation Factor (TF) as ratio of metal concentration in the shoot to the root were calculated using the following formula^61^:

$${\text{BCF}} = \frac{{{\text{Metal~}}\;{\text{level}}_{{{\text{root}}}} }}{{{\text{Metal~}}\;{\text{level}}_{{{\text{water}}}} }}\;\;\;{\text{BAC}} = \frac{{{\text{Metal}}\;{\text{~level}}_{{{\text{shoot}}}} }}{{{\text{Metal~}}\;{\text{level}}_{{{\text{water}}}} }}\,\;\;{\text{TF}} = \frac{{{\text{Metal~}}\;{\text{level}}_{{{\text{shoot}}}} }}{{{\text{Metal~}}\;{\text{level}}_{{{\text{root}}}} }}$$

The rate of metal uptake and elimination by plats were calculated as follows^62^:

$${\text{Rate~}}\;{\text{of~}}\;{\text{metal~}}\;{\text{uptake}} = \frac{{{\text{Metal~}}\;{\text{level}}_{{{\text{exposed}}}} - {\text{~Metal~}}\;{\text{level}}_{{{\text{control}}}} }}{{{\text{Day}}\left( {\text{s}} \right)\;{\text{of}}\;{\text{metal}}\;{\text{~exposure}}\;{\text{~}}\left( {{\text{day}}} \right)}}$$

$${\text{Rate~}}\;{\text{of~}}\;{\text{metal}}\;{\text{elimination}} = \frac{{{\text{Metal~}}\;{\text{level}}_{{{\text{end~}}\;{\text{of~}}\;{\text{metal}}\;{\text{~exposure}}}} - {\text{Metal}}\;{\text{level}}_{{{\text{end~}}\;{\text{of}}\;{\text{metal}}\;{\text{elimination}}}} }}{{{\text{Day}}\;\left( {\text{s}} \right)\;{\text{of~}}\;{\text{metal}}\;{\text{elimination~}}\;\left( {{\text{day}}} \right)}}$$
